# T2Candida assay: diagnostic performance and impact on antifungal prescribing

**DOI:** 10.1093/jacamr/dlad035

**Published:** 2023-04-06

**Authors:** Rita Patrocínio de Jesus, Hamish Houston, Annemiek H J Schutte, Stephen Morris-Jones, Neil Stone, Rebecca Gorton, Gabriele Pollara

**Affiliations:** Department of Clinical Microbiology, University College London Hospitals NHS Foundation Trust, London, UK; Department of Clinical Microbiology, University College London Hospitals NHS Foundation Trust, London, UK; Department of Clinical Microbiology, University College London Hospitals NHS Foundation Trust, London, UK; Department of Clinical Microbiology, University College London Hospitals NHS Foundation Trust, London, UK; Department of Clinical Microbiology, University College London Hospitals NHS Foundation Trust, London, UK; Hospital for Tropical Diseases, University College London NHS Foundation Trust, London, UK; Department of Infection Sciences, Health Services Laboratories, London, UK; Department of Clinical Microbiology, University College London Hospitals NHS Foundation Trust, London, UK; Hospital for Tropical Diseases, University College London NHS Foundation Trust, London, UK; Division of Infection & Immunity, University College London, London, UK

## Abstract

**Objectives:**

To assess the performance of T2Candida for the diagnosis of invasive candidiasis (IC) against gold standards of candidaemia or consensus IC definitions, and to evaluate the impact of T2Candida on antifungal drug prescribing.

**Methods:**

A retrospective review was undertaken of all T2Candida (T2MR technology, T2 Biosystems) performed from October 2020 to February 2022. T2Candida performance was evaluated against confirmed candidaemia or against proven/probable IC within 48 hours of T2Candida, and its impact on antifungal drug prescriptions.

**Results:**

T2Candida was performed in 61 patients, with 6 (9.8%) positive results. Diagnostic performance of T2Candida against candidaemia had a specificity of 85.7% and negative predictive value (NPV) of 96.8%. When comparing T2Candida results with consensus definitions of IC, the specificity and NPV of T2Candida was respectively 90% (54/60) and 98.2% (54/55) for proven IC, and 91.4% (53/58) and 96.4% (53/55) for proven/probable IC. Antifungals were initiated in three of six patients (50%) with a positive T2Candida result. Thirty-three patients were receiving empirical antifungals at the time of T2Candida testing, and a negative result prompted cessation of antifungals in 11 (33%) patients, compared with 6 (25%) antifungal prescriptions stopped following negative beta-d-glucan (BDG) testing in a control population (*n* = 24).

**Conclusions:**

T2Candida shows high specificity and NPV compared with evidence of *Candida* bloodstream infection or consensus definitions for invasive *Candida* infection, and may play an adjunctive role as a stewardship tool to limit unnecessary antifungal prescriptions.

## Background

Invasive candidiasis (IC) complicates the care of patients in ICU receiving immunosuppressive treatments and following complex surgery.^1^ Mortality remains high, approaching 40% in some case series.^2^ Early diagnosis is associated with better outcomes, but its study is complicated by evolving definitions;^3^ imperfect sensitivity of microbiological culture;^4^ and the inability of non-culture-based tests, such as serum 1,3-β-d-glucan (BDG) antigen detection, to distinguish candidiasis from other fungal infections.^5^

T2Candida (T2MR technology, T2 Biosystems, Lexington, MA, USA) is a novel automated molecular method combining PCR with amplification detection by magnetic resonance-based technology.^4^ This assay detects the five most common *Candida* species implicated in IC from whole blood: *C. albicans/tropicalis*, *C. parapsilosis* and *C. krusei*/*glabrata*.^4^ Initial performance data reported a sensitivity and specificity of 91% and 96%, respectively, with a negative predictive value (NPV) of 99.5% for a population with 5% prevalence of candidaemia,^6^ although the study included several artificially spiked positive blood cultures (BCs). Subsequent studies have also observed high NPV (80%–100%), indicating T2Candida can have a role in discontinuing antifungals.^7–9^ It has also been assessed as a tool for early initiation antifungal therapy, with earlier detection of *Candida* compared with BC in complicated candidaemia,^10^ and reduced time to targeted antifungal therapy compared with BC.^11^ However, some studies have reported lower sensitivity (42.8%) for diagnosing IC (with or without candidaemia),^12^ 33.3% for the diagnosis of intra-abdominal candidiasis^13^ and 59% for the diagnosis of proven/probable IC.^9^

We present data from the first UK centre to make this assay routinely available to infection specialists. We sought to assess T2Candida performance for the diagnosis of IC as determined either by candidaemia or by composite microbiological and clinical parameters,^3^ and to explore its impact on real-life clinical management and antifungal prescribing.

## Methods

### Patient population

We included all patients admitted to University College London Hospitals (UCLH), UK, for whom the T2Candida assay was performed between October 2020 and February 2022. As a comparator, a non-overlapping patient population between May 2019 and December 2021 was used to assess the role in antifungal prescribing of BDG, a diagnostic test routinely available during this time for use in any patient at our institution.

### Data collection

We retrospectively collected T2Candida results alongside demographic, clinical (including risk factors for IC), antifungal prescriptions (fluconazole or an echinocandin) and microbiological data (cultures, BDG) from patient records.

### Case definitions

We used consensus IC definitions^3^ of proven IC (culture proven or visualization of *Candida* species in blood or sterile site) or probable IC (at least one clinical plus at least one mycological criterion, plus at least one host factor) (Table S1, available as Supplementary data at *JAC-AMR* Online). We defined ICU admission as attendance for >24 h in critical care during admission; presence of central venous catheter (CVC) as having a central line *in situ* at time of clinical suspicion of IC or T2Candida collection; steroid use as prednisolone equivalent ≥20 mg/day; abdominal surgery and renal replacement therapy (RRT) if either occurred during hospital admission; and neutropenia if present at the time of clinical suspicion of IC or T2Candida collection. Patients receiving antifungals are routinely reviewed on antimicrobial stewardship rounds at our institution. The impact on prescribing was defined as cessation of antifungals for IC at the time of this review that was attributable to negativeT2Candida or BDG result within 5 days of performing this test.

### Statistical analyses

Data were analysed using Stata/SE 17.0. We assessed performance characteristics (sensitivity, specificity, positive predictive value and NPV) of T2Candida compared with isolation of *Candida* species from BC and IC definitions. Where multiple tests had been performed, we included either the first negative test if all tests were negative or the first positive test per patient. The analysis was limited to include T2Candida testing performed within 48 h of the microbiological culture or BDG results.

### Ethics statement

The study was approved by the Audit and Research Committee in the Department of Clinical Microbiology, University College London Hospitals, who stated that as a retrospective review of routine clinical data analysed for service development, further formal ethical approval was not required.

## Results

### Patient demographics

In patients with a T2Candida test, the median age was 55 years (39–68), 40 patients were male (65.6%) and median Charlson comorbidity index was 3 (Table S1). Forty-two patients (69%) were admitted to ICU during admission. Forty-six (75.4%) had a CVC in place. Risk factors for IC included fever unresponsive to antibiotics (37.7%, *n* = 23), steroids (31.2%, *n* = 19), chemotherapy (26.2%, *n* = 16), abdominal surgery (21.3%, *n* = 13), neutropenia (19.7%, *n* = 12) and RRT (19.7%, *n* = 12).

### Prevalence and T2Candida positivity

The prevalence of candidaemia was 8.2% (5/61) at any point during admission. When using consensus definitions of IC,^3^ 13.1% (8/61) and 8.2% (5/61) patients were defined as proven or probable IC cases, respectively, at any point during their admission. Overall, 79 T2Candida tests were performed in 61 patients. Six patients had a positive result (9.8%), of which five identified *C. albicans/tropicalis* and one *C. glabrata/krusei*.

### Diagnostic performance of T2Candida against BC

Thirty-six (59%) patients had a T2Candida performed within 48 h of BC. Only one case of proven IC was not detected by the T2Candida, although one patient with a positive T2Candida result for *C. glabrata* grew not only *C. glabrata,* but also *C. kefyr* and *C. lusitaneae* (Table 1). The specificity and NPV for T2Candida relative to candidaemia was 85.7% (30/35) and 96.8% (30/31), respectively (Table 2).

**Table 1.** Characteristics of patients with positive T2Candida results and/or that underwent T2Candida testing within 48 h of microbiological diagnosis of proven or provable IC

*ID*	*Age, sex*	*Risk factors for IC*	*Bassetti et al.^2^ definitions*	*T2Candida result*	*Candida species*	*Sample with identification of Candida by culture*	*Beta*-*d-*g*lucan (pg/mL)^b^*	*Antifungal when T2Candida performed*	*Antifungal after T2Candida result*	*Clinical presentation*
*1*	17, F	CVC, neutropenia, chemotherapy	No^a^	Positive	*C. albicans/tropicalis*	—	D −3: 178 D +4: 233	Yes	Already on antifungal, no change	Disseminated (brain, renal and liver lesions)—previously proven
*2*	67, M	CVC, abdominal surgery	Probable	Positive	*C. glabrata*	Abdominal fluid^c^ (D 0)^b^		Yes	Already on antifungal, no change	Retroperitoneal collection post-perforation-complicated ERCP
*3*	65, M	ICU, CVC, renal replacement therapy	No	Positive	*C. albicans/tropicalis*	—	D −7: 238 D −2: 157	No	Started due to result	Fever despite antibiotic coverage; admitted for pneumonia
*4*	25, F	ICU, CVC, steroids	No	Positive	*C. albicans/tropicalis*	—	D 0: Neg	No	Started due to result	Translocation in patient with intestinal failure and colitis
*5*	73, F	ICU, CVC, abdominal surgery	No	Positive	*C. albicans/tropicalis*	—	D −2: 290	Yes	Already on antifungal, no change	Fever despite antibiotic coverage; admitted for oesophageal perforation
*6*	56, F	ICU, CVC, steroids	No	Positive	*C. albicans/tropicalis*	—	D 0: 417 D +4: >500	No	Started due to result	Fever despite antibiotic coverage; admitted for COVID-19
*7*	66, F	ICU, CVC	Proven	Negative	*C. albicans*	Blood (D −2)^b^ and urine	D +2: Neg	No	Already on antifungal, no change	Pyelonephritis with candidaemia
*8*	27, M	ICU, CVC, chemotherapy	Probable	Negative	—	No	D −2: 192 D +4: 224	No	Started despite result	Strong suspicion of infective endocarditis by echocardiogram with negative cultures

D, day; ERCP, endoscopic retrograde cholangiopancreatography; F, female; M, male; Neg, negative.

a Previous isolation in blood and urine (Day −7) in relation to T2Candida date, at the time of T2Candida collection BC negative.

b Day (D) count in relation to T2Candida date. If more than two BDG tests performed, we display the tests taken at either side of the T2Candida assay.

c *C. glabrata*, *C. kefyr* and *C. lusitaneae*.

**Table 2.** Diagnostic performance of T2Candida against candidaemia and invasive candidiasis definitions (within 48 h of T2Candida collection)

Diagnosis of IC	T2Candida result	Comparator^a^		Sensitivity (%) [95% CI]	Specificity (%) [95% CI]	PPV (%) [95% CI]	NPV (%) [95% CI]
		Present	Absent				
Candidaemia^b^	Positive	0	5	0	85.7	0	96.8
	Negative	1	30	[0.00–0.00]	[74.28–97.15]	[0.00–0.00]	[91.00–102.55]
Proven invasive candidiasis^c^	Positive	0	6	0	90	0	98.2
	Negative	1	54	[0.00–0.00]	[82.47–97.53]	[0.00–0.00]	[94.83–101.53]
Proven/probable invasive candidiasis^c^	Positive	1	5	33.3	91.4	16.7	96.4
	Negative	2	53	[21.50–45.16]	[84.34–98.42]	[7.31–26.02]	[91.7–101.06]

a The comparator is, in each 2 × 2 table, the diagnostic criterion for IC used as the gold standard (candidaemia, proven IC and proven/probable IC, respectively). PPV. positive predictive value.

b Included patients with BC collected within 48 h of T2Candida testing (*n* = 36).

c As defined as by Bassetti *et al*.,^2^ considered if within 48 h of T2Candida collection. All patients included (*n* = 61).

### Diagnostic performance of T2Candida against IC case definitions

One patient (1.6%) was classified as proven IC, 2 patients (3.6%) were classified as probable IC and 58 patients (94.6%) were controls (Table 1).

- Patient 2 (probable)—Positive T2Candida for *C. glabrata* in parallel with culture-positive abdominal fluid.
- Patient 7 (proven)—Negative T2Candida in parallel with *C. albicans* candidaemia.
- Patient 8 (probable)—Negative T2Candida in parallel with sequential positive BDG results and infective endocarditis (echocardiogram).

T2Candida had specificity and NPV of 90% (54/60) and 98.2% (54/55) for the diagnosis of proven IC, and 91.2% (53/58) and 96.4% (53/55), respectively, for the diagnosis of proven/probable IC (Table 2).

### Impact of T2Candida on antifungal stewardship

Thirty-six patients (59.0%) were receiving systemic antifungal therapy for clinically suspected IC at the time of T2Candida testing. Among the six patients with a positive T2Candida result, three (50%) were already receiving antifungal therapy and T2Candida positivity resulted in initiation of antifungals in the remaining three (50%).

Antifungal therapy was initiated in 3/61 (4.9%) due to a positive T2Candida result, of which two had a subsequent positive BDG test and therefore, without T2Candida, would have initiated antifungal treatment later in their clinical course.

For 55 patients with a negative T2Candida, 33 (60%) were being treated as cases of IC. Negative T2Candida results prompted cessation of antifungal therapy in 11/33 (33%), with no subsequent clinical need to restart antifungals. In a control population receiving antifungal therapy for suspected IC, of which 13/24 (54%) were admitted to ICU during admission, and for whom T2Candida was not available or performed, a negative BDG result prompted cessation of antifungals in 6/24 (25%) of patients.

## Discussion

We present the first UK data on the diagnostic performance and impact of T2Candida on management of patients at risk of IC. In line with other studies^7,8,9,14^, T2Candida demonstrated a high specificity (>85%) and NPV (>95%) informing cessation of antifungal therapy in approximately one-third of patients, demonstrating the utility of T2Candida as an aid to antifungal stewardship (AFS), and a comparable impact to that of a negative BDG result. Diagnostic-driven strategies, especially through non-culture-based testing, can improve AFS,^15^ and contribute to reducing unnecessary antifungal treatment.^7,16^ Given the significant toxicity and cost of antifungals,^17^ the AFS benefits of the T2Candida test may even offset its upfront costs.

We observed a low number of antifungal initiation based on a positive T2Candida alone, consistent with the high specificity observed and with a previous report of lower inappropriate antimicrobial use when T2Candida and T2Bacteria were used to diagnose bloodstream infections.^18^ Due to the low number of confirmed cases of IC, we did not formally report sensitivity, but it was evident that not all IC cases resulted in a positive T2Candida result. Previous studies have indicated higher test sensitivity when compared with proven candidaemia (71%–91%),^6,12^ highlighting the importance of defining test characteristics in different clinical settings and the variability in gold standard definitions used for *Candida* infections. We included patients with proven or probable IC, as opposed to those only with candidaemia, relevant as T2Candida sensitivity is decreased in deep-seated IC compared with candidaemia.^9,13,19^

This study is limited by its retrospective, single-centre nature. We used consensus definitions of IC as a gold standard,^3^ but we acknowledge these were devised for ICU patients and that one-third of our cohort did not require ICU admission. As a novel assay, there is as yet no agreed local or international guideline or criteria for ordering T2MR, and therefore patients for whom individual clinicians chose to request T2Candida may represent a biased sample. Most of the data from the comparator BDG-tested population preceded availability of T2Candida testing, and the asynchronous nature of these populations urges us to caution against a direct comparison of these two tests’ impact. Their combination (e.g. positive BDG followed by negative T2Candida) may yet prove to have the greatest role in AFS, an aspect that could not be explored by our study. Nevertheless, we have demonstrated that T2Candida can support the exclusion of IC in a high-risk population, serving as an adjunctive antimicrobial stewardship tool to aid de-escalation of empirical antifungals. We support further assessment of the impact of T2Candida in multicentre studies on antifungal prescriptions and clinical outcomes.

## Supplementary Material

dlad035_Supplementary_Data

## Supplementary data

Table S1 is available as Supplementary data at *JAC-AMR* Online.
