# Supplementary material for: T2Candida assay: diagnostic performance and impact on antifungal prescribing
Source: JAC Antimicrob Resist. 2023 Apr 6;5(2):dlad035. doi: 10.1093/jacamr/dlad035 (PMC10076932; doi:10.1093/jacamr/dlad035)
Supplement: dlad035_Supplementary_Data [file dlad035_supplementary_data.docx]

**Supplementary Table S1**: Baseline demographics and clinical characteristics of the patients included in the study (n=61)

| Characteristics | n=61 |
| --- | --- |
| Age (years), median (Q1-Q3) | 55 (39-68) |
| Male, n (%) | 40 (65.6) |
| Charlson comorbidity index, median (Q1-Q3) | 3 (2-5) |
| Risk factors for IC, n (%) |  |
| CVC | 46 (75.4) |
| ICU | 42 (68.9) |
| Fever unresponsive to antibiotics | 23 (37.7) |
| Steroids | 19 (31.2) |
| Chemotherapy | 16 (26.2) |
| Abdominal surgery | 13 (21.3) |
| Neutropenia | 12 (19.7) |
| Dialysis | 12 (19.7) |
| Haematopoietic stem cell transplantation | 6 (9.8) |
| Pancreatitis | 2 (3.3) |
| Solid organ transplant | 1 (1.6) |
| Candidaemia throughout admission, n (%) | 5 (8.2) |
| Positive T2Candida, n (%) | 6 (9.8) |
| Proven IC* throughout admission, n (%) | 8 (13.1) |
| Probable IC* throughout admission, n (%) | 5 (8.2) |

* Definitions as described by Bassetti *et al.*^3^:

- (1) proven IC (culture-proven recovery of *Candida* species from blood or sterile sites [including fluid from drains placed within 24 hours of sampling]; or histopathologic, cytopathologic, or direct microscopic examination of material from sterile sites with visualisation of features consistent with *Candida* species)

- (2) probable IC (presence of at least one clinical criterion [ocular findings, hepatosplenic lesions by computed tomography, consistent clinical or radiological nonpulmonary abnormalities], plus at least one mycological criterion [positive BDG in 2 consecutive samples, recovery of *Candida* species in intra-abdominal specimen collected surgically or within 24 hours of drain placement] plus at least one host factor criterion [glucocorticoid treatment, neutrophil abnormality, impaired gut wall integrity, impaired cutaneous barriers to bloodstream infection, *Candida* colonisation, hematopoietic stem cell transplantation, solid organ transplant].
